# Lying Postures of Dairy Cows in Cubicles and on Pasture

**DOI:** 10.3390/ani9040183

**Published:** 2019-04-21

**Authors:** Elaine van Erp-van der Kooij, Osama Almalik, Daniel Cavestany, Judith Roelofs, Frank van Eerdenburg

**Affiliations:** 1 Department of Applied Biology, HAS University of Applied Sciences, PO Box 90108, 5200 MA ‘s Hertogenbosch, The Netherlands; o.almalik@has.nl; 2 Animal Science and Veterinary Medicine, University of the Republic, 18 de Julio 1824, Montevideo, Uruguay; daniel.cavestany@gmail.com; 3 Department of Animal Husbandry and Animal Care, HAS University of Applied Sciences, PO Box 90108, 5200 MA ‘s Hertogenbosch, The Netherlands; j.roelofs@has.nl; 4 Faculty of Veterinary Medicine, Utrecht University, Yalelaan 1, 3584 CL Utrecht, The Netherlands; F.J.C.M.vanEerdenburg@uu.nl

**Keywords:** dairy cattle, lying postures, housing system, orientation

## Abstract

**Simple Summary:**

Cows housed indoors with cubicles are probably more restricted in their choice of lying posture and orientation compared with cows housed on pasture. We therefore studied lying postures on pasture in Uruguay and the Netherlands, and in cubicles in the Netherlands, also recording orientation on pasture in Uruguay and divider and bedding type in Dutch cubicles. Cows on pasture in Uruguay showed more long postures, lying on their belly with the neck stretched, and, per herd, cows preferred a specific lying orientation. Dutch cows on pasture showed more wide postures, lying on their side with three legs stretched, while in cubicles they showed more narrow postures, lying on their side with folded hind legs. More long and less short postures were seen in cubicles with soft floors and English dividers; more narrow postures were seen in cubicles with concrete floors. Wide postures were seen more in cubicles with mattresses and free-hanging dividers. We conclude that since cows in cubicles show more narrow postures than on pasture and cannot choose lying orientation, their choice in showing preferred behavior is restricted. More research is needed to study the consequences of restricted choice in lying behavior on the health and welfare of dairy cows.

**Abstract:**

Cows housed indoors with cubicles are probably more restricted in their choice of lying posture and orientation compared with cows housed on pasture. We therefore compared lying postures on pasture in Uruguay and the Netherlands with lying postures in cubicles in the Netherlands, also recording orientation on pasture in Uruguay and divider and bedding type in Dutch cubicles. We visited one farm with four herds in Uruguay, doing live observations, and 25 Dutch farms, taking pictures of cows. Observations of 205 cows on pasture in Uruguay showed more long postures, lying on their belly with their neck stretched. Two herds preferred lying towards north and south, while one herd preferred west and east. Pictures of 217 cows on pasture in the Netherlands showed more wide postures (lying on the side with three or four legs stretched out). Pictures of 527 cows in cubicles in the Netherlands showed more narrow postures (lying on the side with hind legs folded). More long postures (lying on the belly with a stretched neck) and less short postures (lying with the head folded back) were seen in cubicles with soft floors and English dividers; more narrow postures were seen in cubicles with concrete floors. Wide postures were seen more in cubicles with mattresses and free-hanging dividers. We conclude that since cows in cubicles show more narrow postures than on pasture and cannot choose their orientation, their choice in showing preferred behavior is restricted. More research is needed to study the consequences of restricted choice in lying behavior on the health and welfare of dairy cows.

## 1. Introduction

Since the 1960s, most dairy cows in Europe and the USA have been housed in cubicles. Cubicles are partitioned places for the cows to lie down, separated by dividers. Cubicles have neck rails and brisket boards to keep the cows within the cubicle, and have them urinating and defecating outside the cubicle. Bedding materials within the cubicles can consist of sand or other soft materials, mats or mattresses, or sawdust on concrete floors. In loose housing systems with cubicles, cows enjoy freedom of movement, which is good for animal welfare [1], and they can choose in which cubicle to lie and rest. Cows can freely interact with each other and show social behavior as well as avoid other cows if they want to. However, in most loose housing systems, cubicles are used, which have certain disadvantages. Although cubicles are easier to clean than compost-bedded pack systems, lying down in a cubicle can cause some problems for the cow. Cows have increased in size over the years, and the cubicles are often too narrow in older buildings compared to the width of the cows; if a cow lies on her side, she does not fit completely in the cubicle, causing her to lie against the divider and partly put her legs in the next cubicle. In addition cubicles are mostly designed to be a bit too short for the length of the cows, for reasons of hygiene and to reduce labor: when standing up, the cow will defecate outside of the cubicle and not on the lying area, but when lying down, often her length does not fit inside the cubicle, so either her head sticks out the front or her behind sticks out the back of the cubicle and hangs over the slatted floor. If cubicles are too short and/or too narrow, cows have difficulties lying down or standing up, and it might restrict them in choosing their preferred lying posture. Although lying times, standing up, and lying down of cows have been studied extensively, lying posture has not been studied to a great extent. It seems that on pasture, cows prefer to lie on their left side, while indoors, cows lie with their ventral side against the activity area, where feeding and milking take place [2]. In a study in which cows were housed on a compost bedded pack, cows mainly laid with their heads up (84.6%), 8.8% laid with their heads back, 5.4% laid with their heads on the ground, and only 0.8% laid flat on their sides [3].

Furthermore, in a cubicle, barn cows cannot choose in which direction to lie. It is reported that it matters to the cow in what direction she lies in relation to the magnetic axis of the earth [4,5]. Generally, cattle (and deer) tend to rest and graze north–south oriented. Finally, in cubicles that are too short or narrow, cows are restricted in their choice of lying postures. For example, for the wide and long postures, there might not be enough space available for the cow, and cows might be forced to use more short and narrow postures. All these factors are more important if the cows are kept inside all year long.

Bedding material can affect lying time and ease of lying down, and thus the health and welfare of cows [6]. In a study comparing cows housed in tie stalls, cows housed on soft rubber mats with straw spent more time lying down than cows housed on concrete floors, with shorter and more-frequent lying bouts, while the cows on soft mats with straw seemed less hesitant to change their posture. Cows on concrete showed more swelling of the carpal joints [7].

Our hypothesis is that cows on pasture are not restricted in their choice for a lying posture, and therefore on pasture will lie oriented towards the north and show more long and wide postures than in cubicles, while cows in cubicles might show more narrow and short postures. We expect cubicle design to influence lying postures, with more long and wide postures in more comfortable cubicles. The aims of the present study were (1) to determine the preferred orientation of cows lying on pasture in outdoor systems, (2) to investigate the postures of dairy cows lying on pasture versus in cubicles, and (3) to assess the relationship between cubicle design and lying posture. 

## 2. Materials and Methods 

### 2.1. Dairy Farms and Observations 

**Figure 1:**
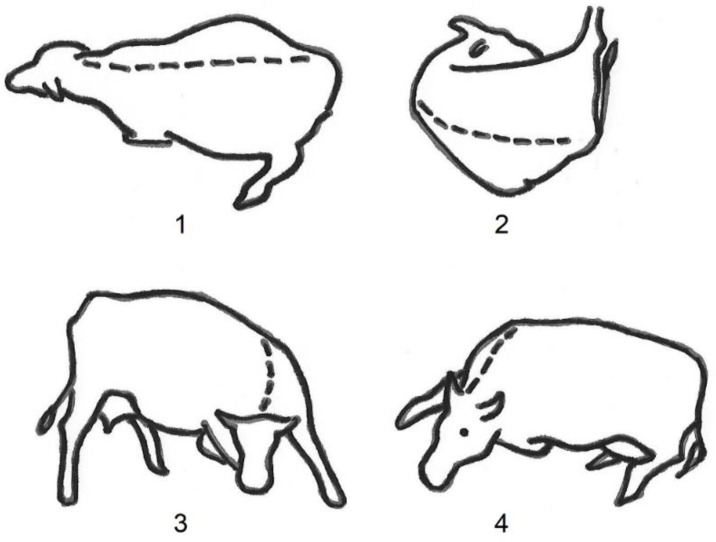
Lying postures of dairy cows: long (**1**), short (**2**), wide (**3**), and narrow (**4**). After [8].

In this study, lying postures were recorded of dairy cows on 26 different farms—25 Dutch farms with a cubicle housing system and pasturing in summer, and one farm in Uruguay where cows were kept on pasture night and day, year-round. All cows were Holstein Friesian. Dutch as well as Uruguayan cows are high-producing animals, with an annual milk yield of 8000–10,000 kg per cow. The Uruguayan farm housed approximately 200 cows, while the Dutch farms varied in size. No selection for high or low of farm size or milk yield of the cows was done. Dutch farms were selected by snowball sampling: Farmers that were willing to cooperate brought in other farms that wanted to join the study. Four lying postures were distinguished (Figure 1, adapted from [8]). In the first posture, “long”, the cow lies on its sternum and ventral side of the abdomen with the neck straightened. In the second posture, “short”, the cow lies on its sternum and ventral side of the abdomen, curled up with the head turned back. In the third posture, “wide”, the cow lies on its lateral side, hind legs stretched. The front legs can be stretched or not stretched. In the fourth posture, “narrow”, the cow lies on its sternum and on its lateral side, hind legs not stretched. The difference between the four postures is the spine of the cow: in the long posture, the spine is upright and the cow lies on its abdomen, while in the narrow posture, the spine is tilted to the side and the cow lies on its ventral side. Each posture can be shown lying on the left or the right side.

Two trained observers did the observations in Uruguay, while fourteen trained observers, consisting of two groups of seven observers each, did the observations in the Netherlands. All observers were trained by one and the same trainer. The training consisted of comparing photographs of lying cows with the pictures of postures as shown in Figure 1. Results of the observers were compared with the trainer. At the start of the training in the Netherlands, a small subset of approximately 10–15 photographs were scored independently by two groups of observers and by the trainer, resulting in kappa values before training of 0.50 and 0.49 for both groups compared with the trainer, a value which is considered “moderate”. These results were discussed with the observers, and scoring of the observers was adjusted according to the scoring of the trainer. After that, observers scored the photographs independently and, when in doubt, discussed results with each other and with the trainer. An inter-observer reliability was not calculated repeatedly during the study. After the study, a subset of 50 photographs of cows on Dutch farms lying indoors as well as outdoors was scored again, this time independently by a veterinarian, resulting in a kappa value of 0.35 for the observers compared with the veterinarian, which is considered “fair”. After the training in Uruguay, the recordings were done by live observation and by one observer at the time. Therefore, no inter-observer reliability could be calculated.

Observations were performed during a period in which most of the cows were lying. In the Netherlands, this was in the morning, between 10 and 12 am, while in Uruguay this was three to four hours after morning milking, which started at 5 am. Lameness or other health parameters were not scored, so as not to disturb the cows when lying down. 

No handling, and minimal disturbance of the cows occurred in this study, so we did not need approval of the work by an ethics committee. Observers did not come within close range of the cows, staying outside of the fence during the outdoor observations and staying off the slatted floors and on the outside of the feeding rack during the indoor observations. All research performed at HAS University of Applied Science was discussed with and approved by the HAS supervisor for animal welfare, on behalf of the Animal Welfare Office Utrecht, in order to comply with national legislation and institutional rules and regulations on animal welfare.

### 2.2. Farm in Uruguay

The study in Uruguay was performed at the Dairy Production Department of INIA (Instituto Nacional de Investigation Agropecuaria), La Estanzuela, Uruguay, a dairy farm with 205 Holstein Friesian cows. This farm had a pasture-based system and was visited several times per day between 8 March and 13 April 2010 by two observers. Data were collected in the pasture during a period of six weeks. Four different herds were observed: herd 1 contained approximately 20 pregnant, non-lactating cows, while herds 2, 3, and 4 were non-pregnant and lactating and consisted of approximately 35 cows, 130 cows, and 20 cows, respectively. 

**Figure 2:**
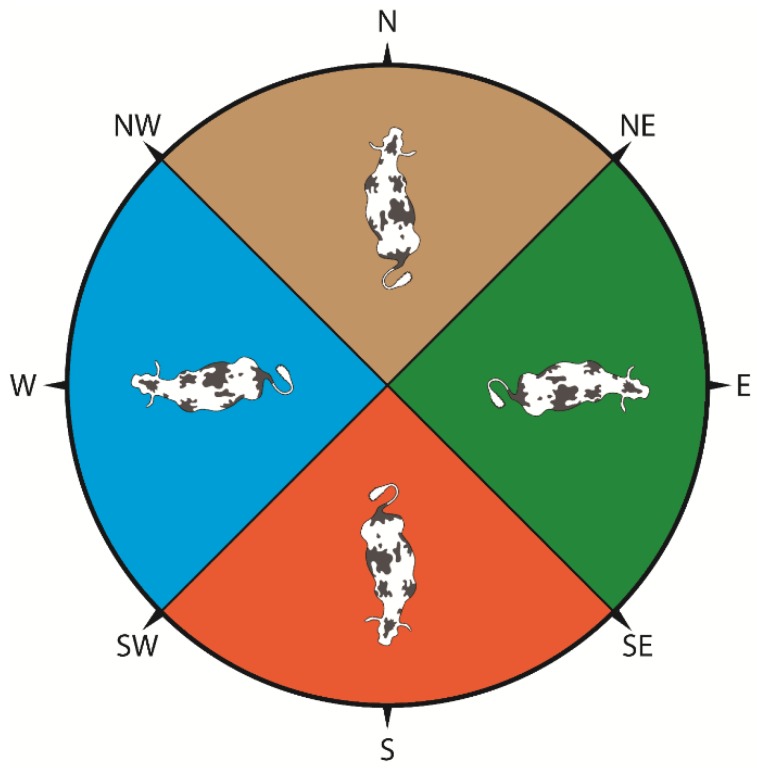
Orientation of cows on pasture in Uruguay. Cows in the northern quarter (beige) with their shoulders between northwest and northeast were scored as “north”; the same principle was used for cows in the eastern quarter (green), scored “east”; in the southern quarter (orange), scored “south”; and in the western quarter (blue), scored “west”. Illustration by van Erp, M.J., 2018 [9].

Cows were observed three to four hours after the start of morning milking (5.00 am.), when most of the cows were lying. The observations were done live, through binoculars, at a distance as far away as possible, and as a result the interference between the observers and the herd was insignificant. The shoulders of the cow were used as reference for determining which direction the cows were facing: north, south, east, or west. When the shoulders were pointing in the northern quarter, “north” was scored, and the same principle was used for the other orientations (Figure 2). 

### 2.3. Dutch Farms

**Figure 3:**
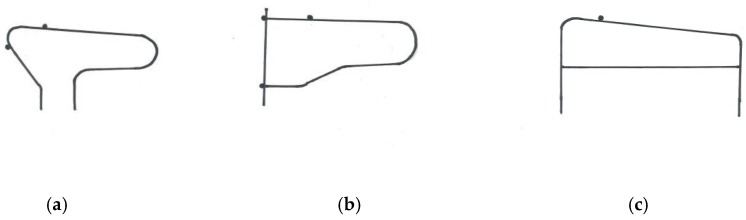
Divider types for dairy cows in cubicles. (**a**) R-shaped divider; (**b**) U-shaped or free-hanging divider; (**c**) English type divider.

In the Netherlands, 25 dairy farms with Holstein Friesian cows participated in the study. The average farm size was 99 ± 43 cows, with a range from 55 to 200 cows. The Dutch farms were visited in two rounds. The first 14 farms were visited once between 17 November 2011 and 19 January 2012, while the cows were housed indoors. During each visit, four hours were spent in the barn recording the postures of dairy cows that were lying down. In this time, the observers made three rounds through the barn to record cow postures. In order not to disturb the cows, the animals were photographed from outside of the feeding fence so that posture was visible, but cow numbers were not recorded. This resulted in a dataset of a varying number of lying postures of cows, with some cows recorded two or three times. Another 11 farms were visited twice between September 2016 and February 2017, once while the cows were on pasture and once while the cows were indoors. During each visit, postures of all lying cows were recorded. Observers again photographed cows from outside the feeding fence in order not to disturb the cows, but this time cow numbers were recorded if visible. Type of bedding material and type of cubicle divider was recorded for the Dutch farms in both rounds. Bedding material was categorized into three categories: soft bedding (deep litter, sand, or straw), hard floor (concrete) with (a thin layer of) sawdust, and mats or mattresses with sawdust. Three categories of cubicle dividers were distinguished: R-shaped dividers, free-hanging or U-shaped dividers, and English dividers (see Figure 3). No information was recorded on brisket boards or neck pipes.

### 2.4. Data Description and Statistical Analysis

Posture data were recorded on one farm in Uruguay with four herds, and on 25 Dutch farms. In Uruguay, herd 1 was visited 10 times, while the other three herds were visited 20 times, resulting in 2116 live recordings of lying postures and orientations of cows on pasture. In 2011–2012, 14 Dutch dairy farms were visited once, resulting in 537 photographs of lying postures of cows in cubicles. In 2016–2017, 11 Dutch dairy farms were visited twice, resulting in 488 photographs of lying postures of cows, 271 in cubicles and 217 on pasture. 

During the Dutch farm visits in 2011–2012, no cow numbers were recorded, and observers walked three times through the barn to take photographs. Therefore, it was uncertain if observers recorded the same cow repeatedly during the same farm visit. That is why, for these data, only the observations of one walking round per visit were retained. We selected the round that resulted in the most recordings, to retain the largest amount of data. This resulted in 262 recordings. During the Dutch farm visits in 2016–2017, cow numbers were read from the neck collars and recorded by the observers (if necessary, binoculars were used on pasture) and only one round of observations was made during each visit; for those data, the observations of unidentified cows were removed, as well as repeated recordings of the same cow on the same day. This resulted in 265 recordings. In Uruguay, cow numbers were also read from the neck collars by the observers, always using binoculars. Recordings of unidentified cows were removed, reducing the data from 2116 to 1306 recordings; furthermore, repeated recordings of the same cows on one observation day were removed; this reduced the data to 357 recordings. In the end, the data added up to 1101 recorded postures—527 in cubicles and 217 on pasture in the Netherlands, and 357 on pasture in Uruguay. 

All 2116 Uruguay records were used to determine the observed lying orientation of the cows. To compare lying posture of the cows indoors and outdoors, and to determine the relation with bedding material and divider type, the data were reduced to unique cow numbers per observation day, to avoid repeated recordings of the same cow.

To determine the preferred orientation of the cows on pasture in Uruguay, a non-parametric one sample chi-square test was used. The null hypothesis was that cows have no preferred orientation, so the observed probability was compared to the hypothesized probability of 0.25 for the four orientations—north, east, south and west. Furthermore, a chi-square test was used to determine whether orientation of the cows differed between the herds. To determine which groups differed from each other, adjusted residuals (ARs) were calculated using a post hoc test [10].

To determine whether outdoor lying postures of the cows on pasture in Uruguay differed within the farm, a non-parametric one-sample chi-square test was used. Again, the null hypothesis was that cows have no preferred lying posture, and therefore the observed probability was compared to the hypothesized probability of 0.25 for the four lying postures of long, short, wide, and narrow. Furthermore, a chi-square test was used to determine whether postures of the cows differed between the herds within the farm. To determine which groups differed from each other, adjusted residuals were calculated using a post hoc test. 

Indoor and outdoor postures for the Dutch cows were compared using a chi-square test. To determine which groups differed from each other, adjusted residuals were calculated as post hoc test.

To determine the relation between lying posture and cubicle properties, a chi-square test was used, where lying postures were compared between cows in cubicles with three cubicle divider types and three types of bedding materials. To determine which groups differed from each other, adjusted residuals were calculated using a post hoc test. This analysis was done only using data from the indoor observations.

All analyses were performed using SPSS Statistics 24 for Windows. Results were considered significant when *p* values were <0.05, and when adjusted residuals (ARs) were >1.96 or <−1.96.

## 3. Results

### 3.1. Orientation of Cows on Pasture in Uruguay

Overall, there were marked differences in the orientations of the cows lying on pasture in Uruguay between the four herds (Pearson’s chi-square = 101.7, degrees of freedom (df) = 9, *p* < 0.000) (Table 1).

When testing preference for orientation within each herd in a one-sample chi-square test with the null hypothesis of equal preferences of 25% per orientation, all herds show significant preferences. Most cows were oriented towards the north in herd 1 (chi-square = 11.3, df = 3, *p* = 0.01) and herd 3 (chi-square = 94.4, df = 3, *p* = 0.000), most cows in herd 4 were oriented towards the north and south (chi-square = 29.2, df = 3, *p* = 0.000), and most cows in herd 2 were oriented towards the west and east (chi-square = 39.0, df = 3, *p* = 0.000).

### 3.2. Lying Postures of Cows on Pasture in Uruguay

**Figure 4:**
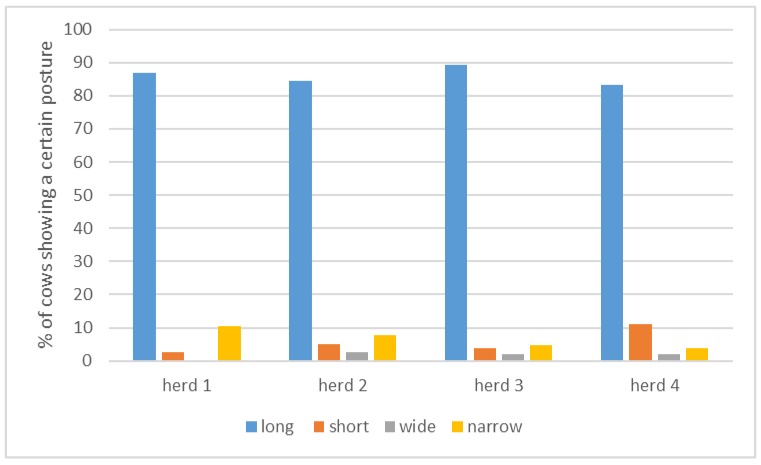
Percentage of “long”, “wide”, “short”, and “narrow” postures per herd, as recorded in 357 observations of 205 cows in four herds on one farm in Uruguay, lying outdoors on pasture.

Lying postures of cows on pasture in Uruguay did not occur with equal probabilities within herds (one-sample chi-square test, *p* < 0.000). Cows showed mostly long postures (*p* < 0.05) in all four herds, with less short, wide, and narrow postures (Figure 4, Table 2). No differences were found in the percentage of lying postures between herds (Pearson’s chi-square = 8.81, *p* = 0.455). 

### 3.3. Lying Postures of Cows in Cubicles and on Pasture in the Netherlands

**Figure 5:**
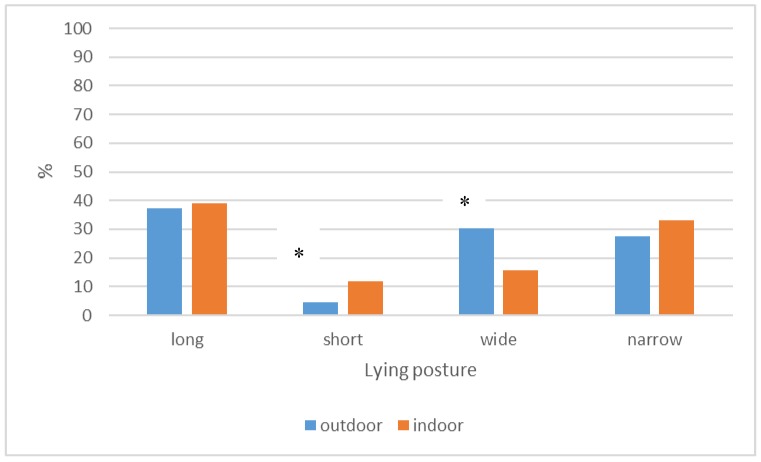
Percentage of “long”, “wide”, “short”, and “narrow” postures as shown by cows lying in indoor cubicles or outdoor on pasture. Results of 744 pictures of cows on 25 Dutch farms in cubicles (n = 527) and on pasture (n = 217). Asterisks indicate significant differences.

When comparing lying postures from cows on pasture (n = 217) and in cubicles (n = 574) in the Netherlands, we found that lying postures differed between indoors and outdoors (Pearson’s chi-square = 26.587, df = 3, *p* < 0.000). Cows in cubicles showed more short postures (AR = 3.1) and cows on pasture showed more wide postures (AR = 4.5). Fully outstretched postures were not seen in the cubicles; however, a posture with both hind legs and one front leg open were also considered a “wide” posture. Indoor and outdoor postures are shown in Figure 5.

### 3.4. Relations with Bedding Type and Type of Cubicle Dividers

**Figure 6:**
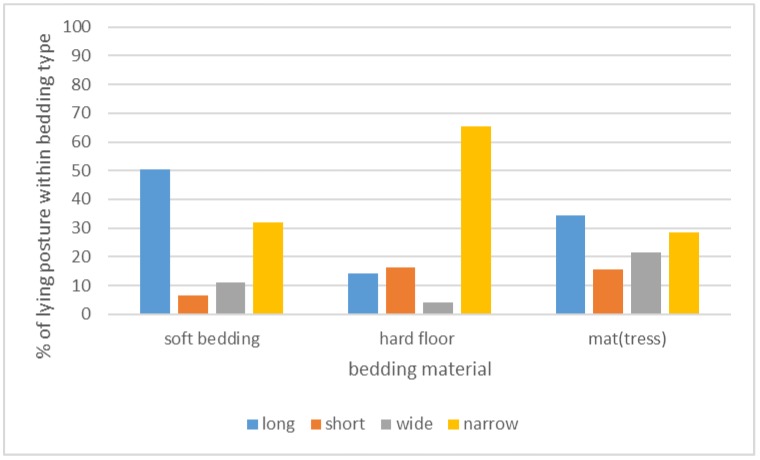
Recorded postures of cows in cubicles with different bedding materials. Results from 527 pictures of lying cows on 25 Dutch farms.

For the Dutch cows lying in cubicles, mostly long (39.1%) and narrow (33.2%) postures were observed, while short (12.0%) and wide (15.7%) postures were seen less often. Relations between lying posture, bedding type, and cubicle dividers were calculated only for the indoor observations on the Dutch farms. Of the 517 observations, 214 (41%) were of soft bedding (deep litter, sand, or straw), 49 (9%) were of hard floors (concrete with sawdust), and 264 (50%) were of mats or mattresses with sawdust. Bedding type was related to the lying postures of the cows (Figure 6) (Pearson’s chi-square = 55.007, df = 6, *p* < 0.001). On soft floors, more long postures were observed (AR = 4.4) and less short (AR = −3.2) or wide (AR = −2.4) postures. On hard (concrete) floors, more narrow postures were observed (AR = 5.0) and less long (AR = −3.7) or wide (AR = −2.4) postures. Finally, on mats or mattresses, more short (AR = 2.7) and wide (AR = 3.7) postures were seen, and less long (AR = −2.2) or narrow (AR = −2.3) postures. 

**Figure 7:**
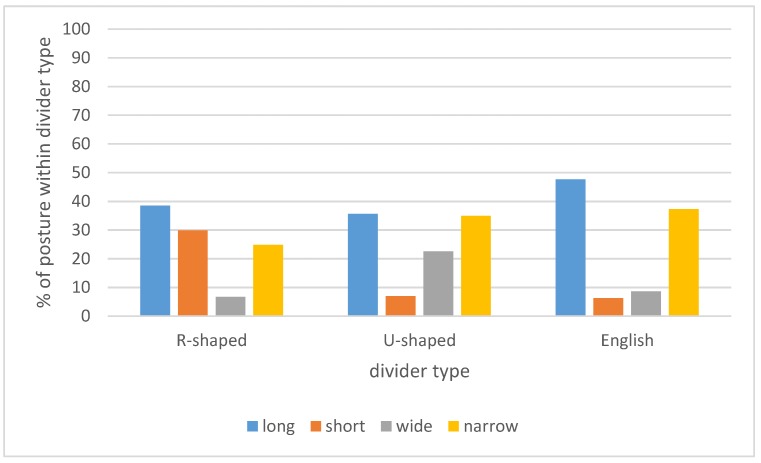
Recorded postures of cows in cubicles with different types of dividers. Results from 527 of lying cows on 25 Dutch farms.

Of the 517 observations, 117 (22%) were of R-shaped cubicle dividers, 284 (54%) were of free hanging or U-shaped dividers, and 126 (24%) were of English dividers. Divider type was related to the lying postures of the cows (Figure 7) (Pearson’s chi-square = 65.387, df = 6, *p* < 0.001). Wide postures were observed more in cubicles with U-shaped dividers (AR = 4.6) and less in cubicles with R-shaped dividers (AR = −3.0) or English dividers (AR = −2.5). Short postures were seen more in cubicles with R-shaped dividers (AR = 6.8) and less with U-shaped (AR = −3.8) or English (AR = −2.2) dividers. Long postures were seen more in cubicles with English dividers (AR = 2.2) and narrow postures were seen less in cubicles with R-shaped dividers (AR = −2.2). 

**Figure 8:**
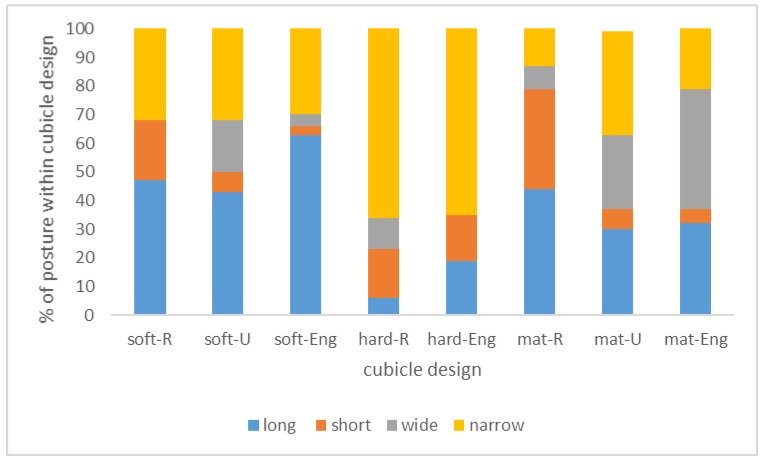
Recorded postures of cows in cubicles with different cubicle designs. Coded combinations of bedding materials and divider types are: soft = soft bedding, hard = hard floor, mat = mats or mattresses; -R = R-shaped dividers; -U = U-shaped dividers; and -Eng = English dividers. Results from 527 lying cows on 25 Dutch farms.

Of the nine possible combinations of bedding material and bedding type, eight were observed: soft floors with R-, U-, or English dividers, concrete floors with R- or English dividers, and mats or mattresses with R-, U-, or English dividers (Figure 8). Lying postures differed between cubicle design types (Pearson’s chi-square = 137.893, df = 21, *p* = 0.000). More long postures were seen on soft floors with English dividers (AR = 4.6), more short postures were seen on mats or mattresses with R-shaped dividers (AR = 6.9), more wide postures were seen on mats or mattresses with U-shaped dividers (AR = 4.4) or English dividers (AR = 3.2), and more narrow postures were seen on hard floors with R-shaped dividers (AR = 3.1) or English dividers (AR = 3.8). 

## 4. Discussion

It was reported [4] that when grazing and resting, cattle align their body axes with a significant preference (70%) for an N–S direction. The authors claim that wind and light can be excluded as a common denominator determining the body axis orientation, but this is not completely true. Light cannot be excluded, because that experiment used satellite images and, therefore, you need sunny weather. The present study found slightly different results. In herds 1, 3, and 4, the north orientation was observed the most, but in herds 3 and 4 an orientation towards the south was also often observed, and in herd 2 we saw cows mostly orientated towards west or east. During the observation weeks, herds were moved from one meadow to another with varying slopes, which may have affected the final results. In a follow-up study, the different slopes should be measured to determine if the measures of the slopes influence a cow’s body direction preference. The differences between the herds, with herd 1 consisting of pregnant and non-lactating cows, and herds 2, 3, and 4 consisting of non-pregnant, lactating cows, cannot explain the differences in preferred orientation.

Cows lying on pasture in Uruguay showed mostly long postures, while cows in the Netherlands showed more wide postures on pasture, and more short postures in cubicles. This might indicate that cows in cubicles are restricted by the limited space of the cubicle, while on pasture they have enough room for long or wide postures. Short or wide postures were observed less than long and narrow postures, and it seems that these are the less-preferred postures for dairy cows. This is in line with a study with cows on compost bedding, where hardly any cows laid flat on their sides [3]. In the same study, a minority (<10%) of the cows showed short postures with their heads back in the lying area, which in our study was demonstrated by 12% of cows in cubicles, and by 4.6–5% of cows on pasture. This suggests that compost-bedded indoor lying areas restrict cows less in their preferred lying behavior than cubicles do, but more than on pasture.

The wide postures that we recorded were defined as both hind legs open and one or two of the front legs open. The completely outstretched posture, with all four legs open, was not seen in the cubicles and only once on pasture in the Netherlands. In Uruguay, no distinction was made between a wide posture with one or with two legs open, so we cannot distinguish between those two postures in the analysis. There might be a difference for the cow between lying with one or with two front legs open; in cubicles, stretching out two front legs is more difficult, if not impossible, especially if there is a brisket board present. This could explain why this posture was not observed in the cubicles. 

It seems that cows are less restricted in their lying behavior when they have a soft surface to lie down on (such as mattresses with sawdust or deep-litter bedding), and when dividers are shaped in a certain way. English-type dividers offer the cows more space to stretch their legs than R-shaped dividers. U-shaped or hanging dividers are supposed to offer the cows even more room to put their legs. The cows did show more wide postures in cubicles with those dividers, but less long postures. 

In Uruguay, live observations were done by one observer, using binoculars. No repeated observations were done, so no intra-observer reliability analysis could be carried out. It would have been better to use two observers and to calculate an inter-observer reliability, but we did not have more observers available at that time. In the Netherlands, photographs were taken and analyzed by multiple observers. A reliability analysis was done at the start of the study. We did not expect the agreement to decrease after the initial moderate agreement (kappa values 0.49 and 0.50) between trainer and observers. Following the first reliability test, no more reliability tests were carried out during the study. When we checked the reliability after the study, however, the agreement between observers and the veterinarian was fair (kappa value 0.35). This was lower than expected. It was not possible to check the inter-observer reliability afterwards, since the observers that carried out the initial coding of the photographs were not available anymore. The reason that the agreement was fair to moderate might be that the different postures were sometimes difficult to distinguish from each other. For example, the long postures, with cows lying on their abdomen, and the wide or narrow postures, with cows lying on their ventral side, might resemble each other when the spine of the cow is in between both postures. This might explain the rather low kappa value between the observers and the veterinarian. For categorizing the pictures, it was important how the spine of the cow was positioned, while in our study we were more interested in whether or not the body and legs of the cow were stretched out and relaxed. For future research, a different set of pictures separating different lying postures for dairy cows might be developed, based on relaxation of the body and legs and not on position of the spine. 

When assessing welfare, the freedom to show natural behavior is one of the criteria for good welfare [11]. In the present study, we showed that cows in cubicles are not able to display certain aspects of their natural behavior, since their lying postures differ significantly from those on pasture. This implies that the choice of showing certain lying postures is restricted in indoor systems with cubicles. We also showed that certain types of bedding and dividers restrict cows less than others; there is a relation between cubicle design and lying postures, which means there is an influence of dividers and bedding. Loose housing systems such as deep litter or compost barns seem to restrict cows less than cubicle systems. However, in loose housing systems without restrictions, more disturbances of lying animals and more social encounters occur, especially when cows are not dehorned [12]. Cows lie down longer in tie stalls than in loose housing systems [13], but when comparing a straw yard to a cubicle system, cows show similar or longer lying times in the straw yard than in the cubicles [14]. Although loose housing systems seem more challenging, especially for low-ranking cows, they also give the cows more choice and control over their environment. However, even in those systems, cows do not show the same behavior as outdoors. The relationship between posture and welfare is yet to be determined, but we argue that cows that have the choice of showing certain postures might have a better welfare. The opportunity to show natural behavior may effectively improve cow welfare in practice and is a promising basis for the design of new husbandry systems [15]. Research is needed on the impact of new and innovative housing systems for dairy cows, such as free range systems and the “cow garden” design [16], on lying behavior and welfare. In those systems, cows can lie down without any restrictions and choose their lying postures and orientation not impeded by dividers.

## Figures and Tables

**Table 1:** Orientation of the body axis of cows lying on pasture from four herds on a farm with 205 cows in Uruguay; north, east, south, or west orientation per herd. Results from 2115 live observations.

Herd	N ^1^	S ^1^	W ^1^	E ^1^	n
1	34.4	20.2	22.3	23.2	233
2	23.4	14.9	29.7	32.0	556
3	37.1	24.8	19.0	19.2	1086
4	35.8	31.3	15.4	17.5	240

^1^ N = north, S = south, W = west, E = east; see also Figure 2.

**Table 2:** Percentage of “long”, “wide”, “short”, and “narrow” lying postures for cows on pasture in Uruguay, within and between four herds on one farm. Herd 1 are pregnant, non-lactating cows, while herds 2, 3, and 4 are non-pregnant and lactating. Results of a chi-square analysis with adjusted residuals (AR).

Posture	Herd 1 % (AR ^1^)	Herd 2 % (AR)	Herd 3 % (AR)	Herd 4 % (AR)
Long	86.8 (4.1)	84.6 (5.6)	89.3 (10.5)	83.3 (4.4)
Short	2.6 (−1.3)	5.1 (−1.0)	3.7 (−2.5)	11.1 (0.8)
Wide	0 (−2.5)	2.6 (−3.0)	2.1 (−5.2)	1.9 (−2.7)
Narrow	10.5 (−1.9)	7.7 (−3.4)	4.8 (−6.6)	3.7 (−3.5)

^1^ if AR > 1.96 then % of lying postures is higher than expected; if AR < −1.96 then % of lying postures is smaller than expected.
